# Draft Genome Sequence of the Endophytic Bacterium *Variovorax paradoxus* KB5, Which Has Antagonistic Activity against a Phytopathogen, *Pseudomonas syringae* pv. tomato DC3000

**DOI:** 10.1128/genomeA.00950-17

**Published:** 2017-09-07

**Authors:** Chi Eun Hong, Sung Hee Jo, Ick-Hyun Jo, Haeyoung Jeong, Jeong Mee Park

**Affiliations:** a Plant Systems Engineering Research Center, Korea Research Institute of Bioscience and Biotechnology, Yuseong-Gu, Daejeon, Republic of Korea; b Department of Biosystems and Bioengineering, KRIBB School of Biotechnology, Korea University of Science and Technology, Yuseong-Gu, Daejeon, Republic of Korea; c Infectious Disease Research Center, Korea Research Institute of Bioscience and Biotechnology, Yuseong-Gu, Daejeon, Republic of Korea; d Department of Herbal Crop Research, National Institute of Horticultural and Herbal Science, RDA, Eumseong, Republic of Korea

## Abstract

*Variovorax paradoxus* KB5, isolated from the inside of *Arabidopsis thaliana* leaves, showed antibacterial activity against the phytopathogen *Pseudomonas syringae* pv. tomato DC3000. Here, we report a draft genome sequence of *V. paradoxus* KB5, which contains a delftibactin-like nonribosomal peptide biosynthetic gene cluster.

## GENOME ANNOUNCEMENT

*Variovorax paradoxus* is a plant growth-promoting endophytic bacterium known for its metabolic versatility, including its degradation of diverse environmental contaminants (1). Previously, we isolated bacteria that have antimicrobial activity against plant bacterial pathogens while they are present in leaves of *Arabidopsis thaliana* plants (2). One of these bacteria, strain KB5, was identified as *V. paradoxus* based on 16S rRNA sequence similarity (2).

The *Variovorax paradoxus* KB5 genome was sequenced using the Illumina HiSeq 2500 platform at the National Instrument Center for Environmental Management at Seoul National University (Seoul, Republic of Korea). In total, 1,465,897,430 paired reads (151 cycles) were generated in a library (average insert size of ∼458 bp) constructed using the TruSeq Nano LT DNA sample preparation version 2. After removing the adaptor sequence and refining the quality using Trimmomatic version 0.32 (3), Khmer version 2.0 was used to filter the reads to remove possible contaminants or errors (4). Out of the 752,036,698 bp of reads (average length, 135.8 bp), the assembly contains 116 scaffolds with 6,890,215 bp and 67.5% G+C content. The maximum scaffold length was 323,762 bp, and the *N*_50_ value was 134,572 bp. Automatic genome annotation by the Rapid Annotations using Subsystems Technology (RAST) server (5) predicted 6,422 coding sequences and 50 RNAs; of these, 48% were assigned subsystems. Although RAST analysis suggested *V. paradoxus* EPS (GenBank accession no. CP002417 [6]) to be the closest neighbor of KB5, the average nucleotide identity for 40 phylogenetic marker genes, calculated using specI (7), was only 93.4%, which was below the cutoff value for allocation into a species cluster.

Predicted secondary metabolites were analyzed with antiSMASH (8). *V. paradoxus* KB5 comprised 10 gene clusters, including a delftibactin-like nonribosomal peptide biosynthetic gene cluster. The secondary metabolite, delftibactin, was identified in a gold-associated bacterium, *Delftia acidovorans*, and is involved in gold biomineralization, which detoxifies soluble gold to protect the microbe (9). In addition, RAST predicted the presence of a mechanism that assembles siderophores (5). Siderophore production by microbes can stimulate plant growth; thus, *V. paradoxus* KB5 has potential as a plant growth stimulator (10). Based on these analyses, KB5 may be useful for agriculture. This genome provides further insight into the genetic and functional characteristics of *V. paradoxus* KB5.

### Accession number(s).

The whole-genome shotgun project was deposited at DDBJ/ENA/GenBank under accession number LVHG00000000. The version described herein is LVHG01000000.
